# A standard variation file format for human genome sequences

**DOI:** 10.1186/gb-2010-11-8-r88

**Published:** 2010-08-26

**Authors:** Martin G Reese, Barry Moore, Colin Batchelor, Fidel Salas, Fiona Cunningham, Gabor T Marth, Lincoln Stein, Paul Flicek, Mark Yandell, Karen Eilbeck

**Affiliations:** 1 Omicia, 2200 Powell Street, Suite 525, Emeryville, CA 94608, USA; 2 Department of Human Genetics and Eccles Institute of Human Genetics, 15 North 2030 East, University of Utah, Salt Lake City, UT 84108, USA; 3 Royal Society of Chemistry, Thomas Graham House, Cambridge, CB4 0WF, UK; 4 EMBL Outstation - Hinxton, European Bioinformatics Institute, Wellcome Trust, Genome Campus, Hinxton, Cambridge, CB10 1SD, UK; 5 Department of Biology, Boston College, 140 Commonwealth Avenue, Chestnut Hill, MA 02467, USA; 6 Ontario Institute for Cancer Research, 101 College St, Suite 800, Toronto, ON M5G0A3, Canada; 7 Department of Biomedical Informatics, Health Sciences Education Building, Suite 5700, 26 South 2000 East, University of Utah, Salt Lake City, UT 84112, USA

## Abstract

Here we describe the Genome Variation Format (GVF) and the 10Gen dataset. GVF, an extension of Generic Feature Format version 3 (GFF3), is a simple tab-delimited format for DNA variant files, which uses Sequence Ontology to describe genome variation data. The 10Gen dataset, ten human genomes in GVF format, is freely available for community analysis from the Sequence Ontology website and from an Amazon elastic block storage (EBS) snapshot for use in Amazon's EC2 cloud computing environment.

## Background

With the advent of personalized genomics we have seen the first examples of fully sequenced individuals [1-9]. Now, next generation sequencing technologies promise to radically increase the number of human sequences in the public domain. These data will come not just from large sequencing centers, but also from individual laboratories. For reasons of resource economy, 'variant files' rather than raw sequence reads or assembled genomes are rapidly emerging as the common currency for exchange and analysis of next generation whole genome re-sequencing data. Several data formats have emerged recently for sequencing reads (SRF) [10], read alignments (SAM/BAM) [11], genotype likelihoods/posterior SNP probabilities (GLF) [12], and variant calling (VCF) [13]. However, the resulting variant files of single nucleotide variants (SNVs) and structural variants (SVs) are still distributed as non-standardized tabular text files, with each sequence provider producing its own idiomatic data files [1-9]. The lack of a standard format complicates comparisons of data from multiple sources and across projects and sequencing platforms, tremendously slowing the progress of comparative personal genome analysis. In response we have developed GVF, the Genome Variation Format.

GVF [14] is an extension of the widely used Generic Feature Format version 3 (GFF3) standard for describing genome annotation data. The GFF3 format [15] was developed to permit the exchange and comparison of gene annotations between different model organism databases [16]. GFF3 is based on the General Feature Format (GFF), which was originally developed during the human genome project to compare human genome annotations [17]. Importantly, GFF3, unlike GFF, is typed using an ontology. This means that the terminology being used to describe the data is standardized, and organized by pre-specified relationships. The attribute specification structure of GFF3 files allows extensibility in specifying feature-specific data for different types of features and it is this extensibility that GVF capitalizes on in defining sequence alteration specific data types. Annotation databases have historically developed different in-house schemas; thus, such standardization is required to ensure interoperability between databases and for comparative analyses.

While there are richer ways of representing genomic features using XML (Extensible Markup Language) and relational database schemas, simple text-based, tab-delimited files have persisted in bioinformatics because they balance human with computer readability. Since its adoption as the basic exchange format, two aspects of GFF3 have emerged as essential for success. First, it must be simple for software to produce and parse; second, its contents need to be typed using terms drawn from an ontology. The first aspect means that humans can easily read and edit files with a text editor and perform simple analyses with command-line software tools. The second aspect not only constrains different database curators to use the same terminologies, but also, because of the formal structure of the ontology, allows automated reasoning on the contents of such a file. It therefore prevents ambiguities and conflicting terminologies. GVF builds upon these strengths of GFF3, adopting GFF3's simple, tab-delimited format; and like GFF3, the contents of GVF files are described using the Sequence Ontology (SO) *- *an ontology developed by the Gene Ontology Consortium [18] to describe the parts of genomic annotations, and how these parts relate to each other [19,20]. Using SO to type both the features and the consequences of a variation gives GVF files the flexibility necessary to capture a wide variety of variation data, while still maintaining unified semantics and a simple file format. For example, GVF files can contain both re-sequencing and DNA genotyping microarray experiment data. In addition, GVF capitalizes on the extensibility of GFF3 to specify a rich set of attributes specific to sequence alterations in a structured way. An added benefit of GVF's compliance with GFF3 is that existing parsers, visualization and validation software, such as those developed by the Generic Model Organism Database (GMOD) project to operate on GFF3 files can be used to manipulate and view GVF files. Thus, the GVF complements existing gene and variant nomenclature efforts [21], and provides a simple ontology-based sequence-centric genome file format linking variants to genome positions and genome annotations.

Below we describe the GVF standard and the various additions we have made to GFF3 and SO to support it. We also briefly describe the conversion of the first ten publicly available personal genomes into GVF format. These GVF files are available for download and for cloud computation. We will refer to these data as the 10Gen dataset. This is provided as a service to the biomedical community as a reference dataset for whole genome comparative analyses and software development. This dataset will hopefully foster the development of new tools for the analyses of personal genome sequences.

## Results

We have extended both the GFF3 specification and SO to allow the rigorous description of sequence variations with respect to a reference genome. The first eight columns of a GFF3 file specify the type and source of a feature, its location on a reference sequence, and optionally a score, strand and phase. These columns of data are incorporated into GVF unchanged. The GFF3 format additionally provides the option to append attributes to a sequence feature using tag-value pairs in the ninth column and it is here that GVF specifies additional structure to annotate sequence alteration specific data (Table 1). Effectively describing sequence variants in this fashion has three prerequisites. First, a standard vocabulary is required for additional tags and values. Second, the vocabulary must be defined in a machine-readable fashion. And finally - in order to facilitate downstream analyses - the relationships between terms used must be formally specified using an ontology. In addition to SO, GVF also allows, but does not require, the use of other ontologies such as the PATO, an ontology of phenotypic qualities [22] and the Human Phenotype Ontology (HPO) [23] to categorize the phenotype of the individual.

**Table 1:** A summary of the tag-value pairs, and their requirement for GVF

Tag	Value	Necessity	Description
ID	String	Mandatory	While the GFF3 specification considers the ID tag to be optional, GVF requires it. As in GFF3 this ID must be unique within the file and is not required to have meaning outside of the file
			**ID = chr1:Soap:SNP:12345;**
			**ID = rs10399749;**
			
Variant_seq	String	Optional	All sequences found in this individual (or group of individuals) at a variant location are given with the Variant_seq tag. If the sequence is longer than 50 nucleotides, the sequence may be abbreviated as '~'. In the case where the variant represents a deletion of sequence relative to the reference, the Variant_seq is given as '-'
			**Variant_seq = A,T;**
			
Reference_seq	String	Optional	The reference sequence corresponding to the start and end coordinates of this feature
			**Reference_seq = G;**
			
Variant_reads	Integer	Optional	The number of reads supporting each variant at this location
			**Variant_reads = 34, 23;**
			
Total_reads	Integer	Optional	The total number of reads covering a variant
			**Total_reads = 57;**
			
Genotype	String	Optional	The genotype of this variant, either heterozygous, homozygous, or hemizygous
			**Genotype = heterozygous;**
			
Variant_freq	Real number between 0 and 1	Optional	A real number describing the frequency of the variant in a population. The details of the source of the frequency should be described in an attribute-method pragma as discussed above. The order of the values given must be in the same order that the corresponding sequences occur in the Variant_seq tag
			**Variant_freq = 0.05;**
			
Variant_effect	[1]String: SO term sequence_variant [2]Integer-index [3]String: SO sequence_feature [4]String feature ID	Optional	The effect of a variant on sequence features that overlap it. It is a four part, space delimited tag, The **sequence_variant **describes the effect of the alteration on the **sequence features **that follow. Both are typed by SO. The 0-based index corresponds to the causative sequence in the Variant_seq tag. The feature ID lists the IDs of affected features. A variant may have more than one variant effect depending on the intersected features
			**Variant_effect = sequence_variant 0 mRNA NM_012345, NM_098765;**
			
Variant_copy_number	Integer	Optional	For regions on the variant genome that exist in multiple copies, this tag represents the copy number of the region as an integer value
			**Variant_copy_number = 7;**
			
Reference_copy_number	Integer	Optional	For regions on the reference genome that exist in multiple copies, this tag represents the copy number of the region as an integer in the form:
			**Reference_copy_number = 5;**
			
Nomenclature	String	Optional	A tag to capture the given nomenclature of the variant, as described by an authority such as the Human Genome Variation Society
			**Nomenclature = HGVS: p.Trp26Cys;**

For Dbxrefs, the format of each type of ID varies from database to database. An authoritative list of databases, their DBTAGs, and the URL transformation rules that can be used to fetch the objects given their IDs can be found at this location [45]. Further details can be found here [46]. In addition, a Dbxref can be given as a stable Uniform Resource Identifier (URI).

**Figure 1:**
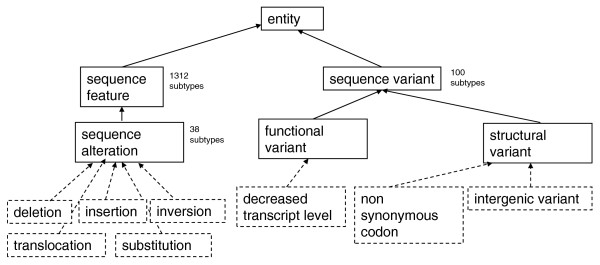
**The top-level terms in the Sequence Ontology used in variant annotation**. There are 1,792 terms in SO, most of which (1,312) are sequence features. There are 100 terms in the ontology that are kinds of sequence variant, of which the two top level terms are shown, and three sub-types, shown with dashed lines, that demonstrate the detail of these terms. The parts of SO that are used to annotate sequence variation files are sequence alteration to categorize the change (five subtypes shown with dashed lines), sequence feature to annotate the genomic features that the alteration intersects, and sequence variant to annotate the kind of sequence variant with regards to the reference sequence.

The SO has been extended in order to describe both the nature of the observed variants and the effects that the variants might have. SO is part of the Open Biological and Biomedical Ontologies (OBO) Library [24], and follows the recommendations and formalisms of the OBO Foundry [25]. This enables machine reasoning across GVF data files using the rich collection of software tools and libraries developed for use with OBO. The key top-level terms are shown in Figure 1. The logic and structure imposed by an upper level ontology means that existing and novel feature annotations are easily added and then immediately computable.

### GVF: a specification for genome variant description

**Figure 2:**
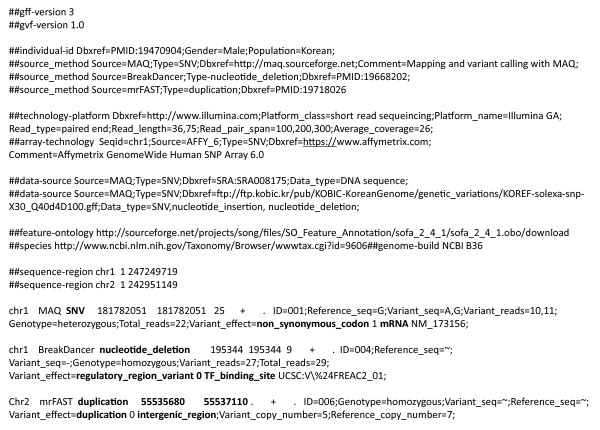
**An example of a GVF annotation, showing three hypothetical sequence alterations: an SNV, a deletion and a duplication**. Lines beginning with '##' specify file-wide pragmas that apply to all or a large portion of the file. Lines are broken over multiple lines and separated by empty lines for presentation in the manuscript, but all data for a given pragma or feature should be contained on one line in a GVF file. A description of the tag-value pairs is given in Table 1.

Figure 2 shows several lines from a typical GVF file. As in GFF3, there are three types of lines: those beginning with '##' specify file-wide pragmas - global features of the genome as a whole; lines beginning with '#' are unstructured comments; and all remaining lines described features of the sequence.

GVF provides nine new pragmas to describe the reference sequence and the methods used to call variants. These pragmas are described in detail in Table 2. The existing genome-build pragma of GFF is mandatory, as all GVF files are dependent on a reference sequence to specify variant positions. While most of the examples discussed here are human genome sequence variants, GVF is a truly generic format. A GVF file can contain sequence variants identified in other organisms as well as identified by DNA microarrays (see example on 10Gen web site for NA_19240). GVF files can also contain variants identified in collections of individuals, as well as population data. The GFF3 species pragma is used to specify other organisms. If one wants to specify multiple individuals in the same file, it is denoted using the source field, and the population_freq tag is provided to describe the frequency of a variant within a population (for example, see the Ensembl database distribution in GVF).

**Table 2:** The pragmas defined by GVF, in addition to those already defined by GFF3 (gff-version, sequence-region, feature-ontology, attribute-ontology, source-ontology, species, genome-build)

Pragma	Allowed tags	Description
file-version	Comment	This allows the specification of the version of a specific file. What exactly the version means is left undefined, but the tag is provided for the case when an individual's variants are described in GVF and then, at a later date, changes to the data or the software require an update to the file. An increment of the file-version could signify such a change. Any numeric version of file-version is allowed
		
file-date	Comment	The file-date pragma is included as a method to describe the date when the file was created. The ISO 8601 standard for dates in the form YYYY-MM-DD is required for the value
		
individual-id	Dbxref, Gender, Population, Comment	This pragma provides details about the individual whose variants are described in the file
##individual-id Dbxref = Coriell:NA18507;Gender = male;Ethnicity = Yoruba; Comment = Yoruba from Ibadan		
		
source-method	Seqid, Source, Type, Dbxref, Comment	This pragma provides details about the algorithms or methodologies used to generate data for a given source in the file. This is used, for example, to document how a particular type of variant was called. A typical use would be to provide a DBxref link to a journal article describing software used for calling the variant data with the given source tag
##source-method Seqid = chr1;Source = MAQ;Type = SNV;Dbxref = PMID:18714091;Comment = MAQ SNV calls;		
		
attribute-method	Seqid, Source, Type, Attribute, Dbxref, Comment	This pragma provides details about algorithms or methodologies for a given attribute tag in the file. This is used to document how a particular type of attribute value (that is, Genotype, Variant_effect) was calculated
##attribute-method Source = SOLiD;Type = SNV;Attribute = Genotype;Comment = Genotype is reported here as determined in the original study		
		
technology-platform	Seqid, Source, Type, Read_length, Read_type, Read_pair_span, Platform_class, Platform_name, Average_coverage. Comment, Dbxref	This pragma provides details about the technologies (that is, sequencing or DNA microarray) used to generate the primary data
##technology-platform Seqid = chr1;Source = AFFY_SNP_6;Type = SNV;Dbxref = URI:http://www.affymetrix.com; Platform_class = SNP_Array;Platform_name = Affymetrix Human SNP Array 6.0;		
		
data-source	Seqid, Source, Type, Dbxref, Data_type, Comment.	This pragma provides details about the source data for the variants contained in this file. This could be links to the actual sequence reads in a trace archive, or links to a variant file in another format that have been converted to GVF
##data-source Source = MAQ;Type = SNV;Dbxref = SRA:SRA008175;Data_type = DNA sequence;Comment = NCBI Short Read Archive http://www.ncbi.nlm.nih.gov/Traces/sra;		
		
phenotype-description	Ontology, Term, Comment	A description of the phenotype of the individual. This pragma can contain either ontology constrained terms, or a free text description of the individual's phenotype or both.
##phenotype-description Ontology = http://www.human-phenotype-ontology.org/human-phenotype-ontology.obo.gz;Term = acute myloid leukemia;Comment = AML relapse;		
		
ploidy	Ontology, Term, Comment	This pragma defines the ploidy for a given genome. This pragma can contain either ontology constrained terms, or a free text description of the individual's ploidy. It is suggested that ontology constrained terms use a subtype of the term PATO:0001374, which includes haploid, diploid, polyploid, triploid etc
##ploidy chr22 1 49691432 diploid		
##ploidy chrY 1 57772954 haploid		

The pragmas defined by GVF may refer to the entire file or may limit their scope by use of tag-value pairs. For example, if a pragma only applies to SNVs that were called by Gigabayes on chromosome 13, then the tags: *Seqid = chr13;Source = Gigabayes;Type = SNV *would indicate the scope. The Dbxref tag within a GVF pragma takes values of the form 'DBTAG:ID' and provides a reference for the information given by the pragma whether that be the location of sequence files or a link to a paper describing a method. Tags beginning with uppercase letters are reserved for future use within the GFF/GVF specification, but applications are free to provide additional tags beginning with lower case letters.

Each of the rows in a GVF file describes a single variant from an individual or population. Each such variant is typed using the SO terms that can describe SNVs, any size of nucleotide insertion or deletion, copy number variations, large structural variations or any of the 38 terms currently related to sequence alterations in SO. In the case of a seemingly complex variation, such as an SNV located within a translocation, each sequence alteration is annotated relative to its location on the reference genome, on a separate line in the file.

The most flexible part of a feature description in GFF3 is the ninth column, where attributes of a feature are given as tag-value pairs (Table 1). It is here that GVF provides additional structure specific to sequence alteration features. Like GFF3, the attribute tag-value pairs in GVF can come in any order. Multiple tag-value pairs are separated from each other by semicolons, tags are separated from values by '=', and multiple values are comma delimited. GVF includes the tags specified by the GFF3 specification, such as ID, Name, Alias, and so on, and in addition 11 additional tags that allow for the annotation of sequence alteration features and constrains the values for some of those attributes to portions of the SO. For example, the sequence of the variant as well as the reference sequence at that position are specified by Variant_seq and Reference_seq tags, respectively. In the case of sequence-based variant calling methods, the number of reads supporting the variant can be given by the Variant_reads tag. The genotype at the variant locus is specified with the Genotype tag. Other features annotated on the genome (gene, mRNA, exon, splice site, transcription start site, and so on) that intersect the variant, along with the effect that the variant has on the feature, are annotated with the Variant_effect tag. For variant sequences that involve deletion or duplication of large regions of the reference sequence, the copy number of the region may be given with the Variant_copy_number tag. Table 1 provides the details for the tags discussed here and the allowed values.

While a great deal of personal genome variation data today comes from next generation sequencing technologies, the GVF standard can also be used to describe variant data from any source creating DNA variation data with nucleotide resolution, including genotyping DNA microarrays, comparative genomic hybridization (CGH) arrays, and others.

Because GVF is a fully compliant extension of GFF3, GVF files provide a basis for exploration and analysis of personal genome sequences with the widely used Bioperl [26], and GMOD toolkits [27]; variant annotations can be viewed by browsers such as GBrowse [28], JBrowse [29], Apollo [30], and analyzed, for example, using the Comparative Genomics Library (CGL) [31]. This means that a GVF file can be passed through a series of analyses, each step adding various attributes to the file, allowing a GVF file to grow progressively richer with each analysis. Complete documentation is available from the website [14].

### A reference personal genomes dataset - '10Gen'

Gold standards and reference datasets are invaluable for software development, testing and for benchmarking the performance of algorithms and tool sets. Classic examples in genomics include the CASP (Competitive Assessment of Protein fold recognition) workshop and its datasets for protein structure comparisons [32,33], the GASP (Genome Annotation Assessment in *Drosophila melanogaster*) [34], EGASP (ENCODE Genome Annotation Assessment Project) [35,36], and NGASP (Nematode Genome Annotation Assessment Project) [37] datasets for gene finding and genome annotation, and the Eisen *et al. *[38] gene expression dataset for microarray analyses. As proof-of-principle for the GVF standard and to facilitate personal genome analyses and the development of software for such analyses, we have parsed the original variant files for ten publicly available personal genome sequences and assembled their variant information in GVF format (Table 3). These ten genomes come from diverse ethnic backgrounds and were produced using a variety of sequencing platforms. Also included in the dataset is a single genome (NA_18507) sequenced with two different technologies. For the genome NA_19240 we present the published DNA genotype microarray data (HumanHap550) variants in gvf format as an additional file. These features of the GVF dataset mean that it is an ideal test dataset for a wide array of anthropological analyses, technical comparisons of sequencing platforms, and eventually personal health analyses. The source data for each GVF file is given in the methods section.

**Table 3:** A reference GVF dataset for public use

Filename	Individual	Ethnicity	Platform	Reference
10Gen_NA19240_SNV	NA19240	African	Life SOLiD	[9]
10Gen_NA18507_ILMN_SNV	NA18507	African	Illumina	[4]
10Gen_NA18507_SOLiD_SNV	NA18507	African	Life SOLiD	[3]
10Gen_Chinese_SNV	Chinese	Asian	Illumina	[5]
10Gen_Korean_SNV	Korean	Asian	Illumina	[6]
10Gen_Venter_SNV	Venter	Caucasian	Sanger	[1]
10Gen_Watson_SNV	Watson	Caucasian	Roche 454	[2]
10Gen_NA07022_SNV	NA07022	Caucasian	CGenomics	[8]
10Gen_NA12878_SNV	NA12878	Caucasian	ABI SOLiD	[9]
10Gen_Quake_SNV	Quake	Caucasian	Helicos	[7]

## Discussion

To fulfill the promise of personal whole genome sequencing it will be critical to compare individual genomes to the reference genome and to one another. One lesson learned from comparative genomics analyses [31-34,37] is that accurate and easy comparisons require a standardized data format. Without a data standard, ambiguities and misunderstandings poison comparative analyses. The GFF3 standard has been widely embraced by the model organism community as a solution to these problems. GVF will provide the same benefits for personal genomics. Although some of the variant file formats currently in use [1-8] and VCF [13] are GFF3-like in spirit, none is a formal extension of GFF3, meaning that their terminologies (tags) are not formally defined, versioned, maintained or OBO compliant [25]. GVF also differs from existing formats in matters of scope. First, GVF is not limited to re-sequencing applications; it also can be used to describe DNA genotyping chip experiments, re-sequencing and DNA-chip data can even be combined in a single file. Second, GVF provides more than just a means to describe how and why a variant was called; it provides an extensive terminology with which to describe a variant's relationship to *- *and impact upon *- *other features annotated on a genome.

Rigorously grounding GVF upon the GFF3 specification has many other benefits as well. Because both file formats are typed using the SO, GFF3 and GVF files can be used together in a synergistic fashion. Moreover, because GVF is a formal extension of the GFF3 standard, existing parsers, visualization tools and validation software, such as those developed by the GMOD project [16] to operate on GFF3 files, can used to manipulate and view GVF files. This will provide enormous benefits for those seeking to analyze personal human genomics data.

In order to jumpstart such analyses, we have also manufactured a reference dataset of variants from ten personal genomes, the 10Gen dataset. These genomes represent a diverse assortment of ethnicities, and were produced using a variety of sequencing platforms. Our hope is that the 10Gen dataset will be used as a benchmark for personal genomics software development, following in the footsteps of other successful benchmark datasets, such as those used by CASP [32,33] for protein structures, GASP/EGASP/NGASP [34,35,37] for gene structures, and Eisen/MIAME (Minimum Information about a Microarray Experiment) [38-40] for gene expression, to name just a few. Moreover, the simplicity of the GVF file format combined with the rigor of its formal specification make GVF ideal for adoption by technology providers, genome centers, population geneticists, computational biologists, evolutionary biologists, health care providers, and clinical testing laboratories.

## Materials and methods

### Extensions to the Sequence Ontology

Using OBO-Edit [41] the SO was extended in three areas: sequence_alteration, sequence_feature and sequence_variant. There are 38 terms to represent the kinds of sequence alteration, 1,283 terms to represent features intersected by the alteration and 100 terms to represent the variant caused by a sequence alteration, such as intergenic_variant and non_synonymous_codon (see the MISO Sequence Ontology Browser on the SO website [42] for complete details).

### Variant files for ten genomes

The variant files from the ten genomes were downloaded from web sites indicated in the references listed in Table 3. These files were converted to GVF format and were manually spot checked for consistency with annotations on the UCSC Genome Browser. They were then analyzed with a genome variation software pipeline that provided additional quality and consistency checks with respect to the NCBI build 36 of the human genome assembly and with data in the dbSNP and OMIM (Online Mendelian Inheritance in Man) databases.

The GVF standard can also be used to describe genotyping DNA microarray-based variant calls. This flexibility means that a single parser can process variant files from both sequencing and DNA genotyping microarray experiments; moreover, because these fields are attributes of the variant, not the file, a single GVF file can contain variants from heterogeneous sets of sequencing and microarray platforms.

### Data downloads

The 10Gen dataset is available for download [43]. Each variant file is named as denoted in Table 3 and additional details are documented in a README file within the download directory. In addition, a cloud compatible version of the data is available as an Amazon elastic block storage (EBS) snapshot [44]. Details for using the snapshot are available from the 10Gen website [43]. This set provides a standard reference dataset and a means to benchmark new analysis procedures. GVF files are also available for download of variant data from Ensembl.

## Abbreviations

CASP: Competitive Assessment of Protein fold recognition; EGASP: ENCODE Genome Annotation Assessment Project; GASP: Genome Annotation Assessment in *Drosophila melanogaster*; GFF: General Feature Format; GFF3: Generic Feature Format version 3; GMOD: Generic Model Organism Database; GVF: Genome Variation Format; NCBI: National Center for Biotechnology Information; NGASP: Nematode Genome Annotation Assessment Project; OBO: Open Biological and Biomedical Ontologies; SNP: single nucleotide polymorphism; SNV: single nucleotide variation; SO: Sequence Ontology; VCF: Variant Call Format.

## Authors' contributions

MGR, MY and KE conceived the project. BM, CB, FS, LS, KE developed technical aspects. BM maintains the GVF specification and data repository. All authors contributed intellectually to the development of the project. MGR, MY and KE wrote the manuscript with input from the authors.
